# Assessment of Active Pharmaceutical Ingredient Particle Size in Tablets by Raman Chemical Imaging Validated using Polystyrene Microsphere Size Standards

**DOI:** 10.1208/s12249-013-0064-9

**Published:** 2014-01-11

**Authors:** Atsushi Kuriyama, Yukihiro Ozaki

**Affiliations:** 1 Technology Research & Development Division, Dainippon Sumitomo Pharma Co., Ltd., 3-45, Kurakakiuchi 1-Chome, Ibaraki-shi, Osaka 567-0878 Japan; 2 Department of Chemistry, School of Science and Technology, Kwansei-Gakuin University, Sanda-shi, Hyogo Japan

**Keywords:** binarization threshold, image analysis, particle size, polystyrene microspheres, Raman chemical imaging

## Abstract

Particle size is a critical parameter for controlling pharmaceutical quality. The aim of this study was to assess the size of the micrometer-scale active pharmaceutical ingredients (API) in tablets using Raman chemical imaging and to understand the effects of formulation on particle size. Model tablets containing National Institute of Standards and Technology traceable polystyrene microsphere size standards were developed to determine the binarization threshold value of Raman chemical images for API particle sizing in specific formulations and processes. Three sets of model tablets containing 5, 10, and 15 μm polystyrene microspheres, used to mimic API, were prepared using a commercial tablet formulation (Ebastel tablets, mean API particle size was about 5 μm). Raman mapping with a 50× objective (NA, 0.75) was applied to tablet cross-sections, and particle size of polystyrene microspheres was estimated from binary images using several binarization thresholds. Mean particle size for three sets of polystyrene microspheres showed good agreement between pre- and postformulation (the slope = 1.024, *R* = 1.000) at the specific threshold value ((mean + 0.5σ) of the polystyrene-specific peak intensity histogram), regardless of particle agglomeration, tablet surface roughness, and laser penetration depth. The binarization threshold value showed good applicability to Ebastel tablets, where the API-specific peak intensity histogram showed a pattern similar to that of polystyrene microspheres in model tablets. The model tablets enabled determination of an appropriate binarization threshold for assessing the mean particle size of micrometer-scale API in tablets by utilizing the unique physicochemical properties of polystyrene microspheres.

## INTRODUCTION

The efficacy of pharmaceutical tablets depends on both the physical and chemical properties of the active pharmaceutical ingredient (API). Particle size, shape, and solid-state form of the API and its spatial distribution in the excipient matrix control the dissolution rate, bioavailability, and stability of the final product (1–5).

In pharmaceutical production, the size of ingredient particles is a critical process parameter for ensuring product quality. This size is determined by techniques such as microscopy and image analysis (4–9), sieving (5,9), laser diffraction (4–6,8–11), dynamic light scattering (4,5,7,12), electrical zone sensing (coulter counter) (4,5,8,10), time-of-flight measurements (4,6,11), and cascade impaction (4,13). The API is often micronized prior to or during the formulation process, and its size is tightly controlled. However, API size after micronization does not always reflect its size in a complex dosage matrix. The API particles undergo physical stresses during formulation processes such as mixing, granulation, drying, blending, tableting, and coating, and even during storage of the final product, depending on its formulation and packaging. However, API particle size in multiple components cannot be specifically assessed using the aforementioned devices. Thus, determination of API particle size in the excipient matrix of the final product is a major challenge in pharmaceutical development.

Recently, vibrational spectroscopy (infrared (IR), near-infrared (NIR), and Raman) has been widely used as a powerful tool in pharmaceutical production to analyze pharmaceutical products and to monitor pharmaceutical processes (14–20). Vibrational spectroscopy requires little or no sample preparation; can be used to characterize functional groups; and is fast, nondestructive, accurate, and economical. Vibrational spectroscopic imaging is also used in chemical imaging (CI) for pharmaceutical production. Using this technique, imaging and vibrational spectroscopy are integrated to obtain both spatial and spectral information on pharmaceutical products (21–28). The high selectivity of vibrational spectroscopy for functional groups means that the concentration and distribution of ingredients can be examined. Thus, even though pharmaceutical formulations and their processes involve multiple component systems, API particle size and its distribution (uniformity) in the excipient matrix are specifically assessed using spectroscopy and chemical imaging (14,25,27,28).

Several studies have reported the application of Raman chemical imaging (Raman CI) for assessing API particle size in pharmaceutical formulations (25,30,31). A number of well-resolved fundamental intra- and intermolecular stretching and bending modes are typically observed in API Raman spectra. Raman CI is suitable for establishing the chemical identity of API particles that have a specific Raman peak. Several factors contribute to the superiority of Raman CI for characterizing APIs in the excipient matrices of tablets (29,30). Henson and Zhang reported characterizing drugs in low-dosage pharmaceutical tablets using a Raman line mapping instrument (30). Particle size and spatial distribution of low dosage (0.5 wt.%) APIs in the tablet matrix were estimated using API binary images from Raman maps. The estimated API particle size was dependent on the particle size distribution of the ingoing API material. Doub *et al.* used wide-field Raman CI to assess the API-specific particle size in nasal spray formulations (31). A Raman imaging particle size distribution protocol was developed and validated by using polystyrene microsphere size standards. Good statistical agreement was obtained between the reported and estimated sizes of the polystyrene particles by Raman CI. However, few studies have rigorously estimated the binarization threshold values for image analysis of pharmaceutical formulations; the values reported have arbitrarily influenced quantitative particle sizing in multiple component systems (26,30,31).

In this study, we investigated the use of Raman CI for sizing micrometer-scale API particles in a pharmaceutical tablet in order to better understand the effects of formulation on API particle size. Model tablets were developed in order to establish an appropriate binarization threshold value for image analysis of multiple component systems. Polystyrene microsphere size standards were used as targets for API particle sizing in the model tablets. These tablets had the same excipient matrix as commercial tablets (Ebastel tablets). Polystyrene particle size standards (hereinafter, PS microspheres) are designed for calibrating particle size analyzers and are traceable to the U.S. National Institute of Standards and Technology (NIST). Here, the NIST traceable PS microspheres acted as internal size standards in the model tablets for Raman particle sizing. Application of Raman CI to the model tablets provided a quantitative basis of the threshold value for assessing the mean particle size of the API in the Ebastel tablets.

## MATERIALS AND METHODS

### Materials and Tablet Preparation

**Fig. 1:**
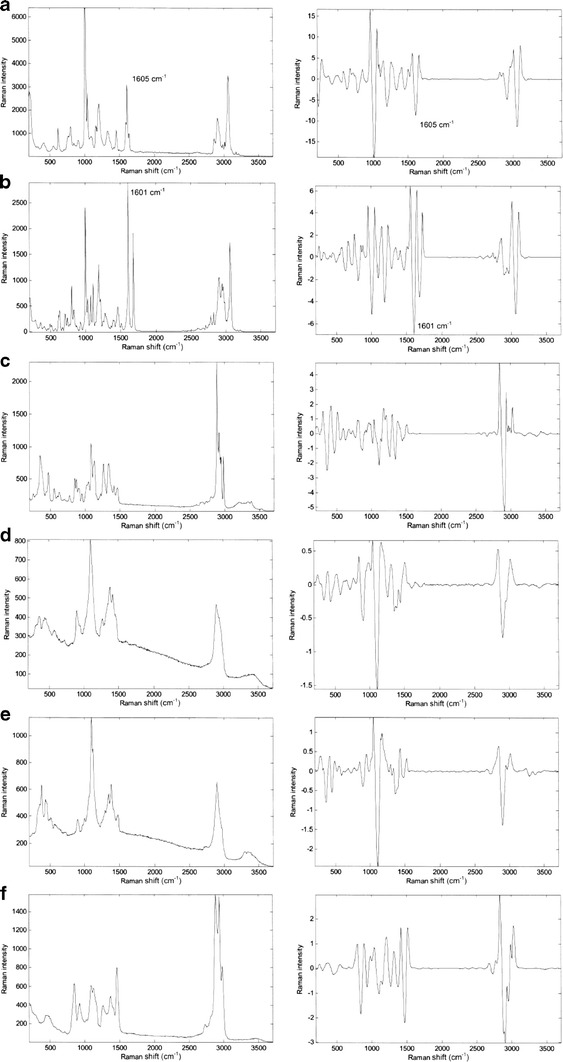
Raman spectra of tablet components (*left*) and second derivatives of the spectra (*right*). **a** PS microspheres, **b** ebastine, **c** lactose, **d** carmellose calcium, **e** crystalline cellulose, and **f** hydroxypropyl cellulose

Ebastel, a widely used antihistamine, was selected as the model drug formulation. Ebastel tablets were prepared according to a commercial process. The API of Ebastel tablets, ebastine (6.25 wt.%), was micronized prior to tablet formulation. Commercial ebastine (Dainippon Sumitomo Pharma, Osaka, Japan) was used to obtain the Raman signature of the pure ingredient (Fig. 1). Pure excipients were also examined to obtain their Raman signatures. The inactive ingredients were lactose (DMV-Fonterra Excipients, Goch, Germany), carmellose calcium (Nichirin Chemicals, Hyogo, Japan), crystalline cellulose (Asahi Kasei Chemicals, Tokyo, Japan), hydroxypropyl cellulose (Nippon Soda, Tokyo, Japan), magnesium stearate (Taihei Chemicals, Osaka, Japan), and light anhydrous silicic acid (Nippon Aerosil, Tokyo, Japan). Three sizes of NIST traceable PS microspheres with mean particle diameters of 5, 10, and 15 μm (Duke Scientific, Palo Alto, CA, USA) were used in the model tablets. The certificate stated that the precise mean particle diameters of the polystyrene particles were 4.9 ± 0.4, 9.8 ± 0.5, and 15.8 ± 0.6 μm as measured by optical microscopy. Three types of model tablets were prepared according to the Ebastel tablet formulation process, using the polystyrene particles instead of ebastine.

### Preparation of Tablet Cross-sections

Tablet cross-sections were prepared for scanning electron microscopy (SEM), 3D confocal laser microscopy, and Raman microscopy. Each tablet was secured in an epoxy resin solution, and the resin was then cured. The tablet was microtomed (Ultracut UCT, Leica, Wetzlar, Germany) to expose its interior and produce a smooth, flat surface.

### Instrumentation

SEM images were acquired under high vacuum on a scanning electron microscope (SU1510, Hitachi High-Tech, Tokyo, Japan). The accelerating voltage was 5.00 kV, and the working distance was 15.0 mm. The sample was sputter coated with Au/Pd using a vacuum evaporator.

The 3D tablet surface was visualized with a 3D measuring laser microscope (LEXT OLS4000, Shimadzu, Kyoto, Japan). Image processing was performed using LEXT software (Shimadzu). Image size was 130 × 130 μm with 1,024 × 1,024 pixels. The diameter of the laser spot was approximately 0.4 μm, using a 100× objective and a numerical aperture (NA) of 0.95. The laser input was 1 mW, and the wavelength was 405 nm. After data collection, images were leveled to remove the slope caused by tilting of the tablet surface.

Raman spectra were collected using a Raman microscope (LabRAM; ARAMIS, Horiba, Kyoto, Japan) and LabSpec 5 Software (Horiba, Kyoto, Japan). Raman scattering was achieved by delivering He-Ne laser (633 nm) excitation of up to 17 mW through a 50× objective with a NA of 0.75 to generate a laser spot diameter of approximately 1 μm. An edge filter was used for Rayleigh light rejection. A 300 grooves/mm dispersive grating combined with a thermoelectrically cooled, silicon charge-coupled device detector achieved a spectral resolution of approximately 4 cm^−1^.

The spectrometer was calibrated each day in reference to the 520 cm^−1^ Raman band of a silicon wafer. After calibration, grating was held in a fixed position throughout data collection.

A static Raman spectral window of 3,700–200 cm^−1^ was used. A motorized XY stage allowed the samples to be scanned with a 1-μm step size under the excitation beam in order to build an array of spectra spanning a two-dimensional area (pixels). The tablet spectrum acquisition time per pixel was typically 0.5 s. Data acquisition covered an area of 10,000 μm^2^ (100 × 100 μm), which consisted of 10,000 spectra with a total acquisition time of nearly 2 h. Three areas in the cross-section of each tablet were examined in this study.

Particle size measurements were carried out on ebastine prior to formulation. The ebastine particle size was measured at a magnification of 50× in a dry powder dispersion particle image analyzer (Morphologi G2; Malvern Instruments, Malvern, UK). Powder was also analyzed using a flow particle image analyzer (FPIA-3000S; Malvern Instruments) with 0.1% neutral surfactant in water as a suspending agent after 30 s sonication. Suspensions were stirred at 300 rpm. Ebastine particle size was measured at a magnification of ×10.

### Raman Particle Sizing

Raman spectral data were analyzed using ISys 4.0 CI software (Malvern Instruments). The Raman particle sizing procedure after data acquisition consisted of the following steps: (1) data preprocessing, (2) data analysis and feeding the results back into the Raman chemical images, (3) creating binary images at the threshold value, and (4) particle size analysis.

Spectra for each pixel were subjected to second-derivative preprocessing (Savitzky-Golay, 31-point width) to remove baseline fluctuations and to improve the image contrast and fidelity, and data analysis was then performed by univariate analysis using a specific Raman peak intensity at one particular wavenumber channel. Many API molecules show specific Raman peaks, which allow them to be distinguished from excipients. By this means, Raman chemical images were produced.

Figure 1a–f displays Raman spectra and the corresponding second derivatives for PS microspheres, ebastine, lactose, carmellose calcium, crystalline cellulose, and hydroxypropyl cellulose. The peak at 1,605 cm^−1^ in the second-derivative spectrum provided the best discrimination between the PS microspheres and the excipients. For ebastine, the peak at 1,601 cm^−1^ was specific and the peak at 1,601 cm^−1^ achieved similar intensity as the PS microsphere peak at 1,605 cm^−1^, at unit exposure time. In this study, Raman chemical images were acquired using the specific peaks at 1,605 cm^−1^ for PS microspheres and 1,601 cm^−1^ for ebastine. These peaks were also used for binary imaging with binarization thresholds.

For particle size analysis, each particle was approximated to a circle that occupied the same area as the particle. The particle size calculated from the pixels was based on the mean equivalent particle diameter, represented by the mean diameter of its corresponding approximated circle.

### Binarization Threshold

Binarization is a critical step for image analysis as the generated binary image with the binarization threshold value influences particle sizing results. In binarization, pixels with a peak intensity at or above the threshold value were set to 1 and were considered as the object, whereas pixels with a peak intensity below the threshold value were set to 0 and were not considered to be the object. Binary images are projected two-dimensionally with clear separation between the object and the background.

Spectroscopic discrimination was observed in this study, as mentioned above; both PS microspheres and ebastine provided a negative peak in the second derivatives, whereas other ingredients showed no peaks. These provided an opportunity to develop a binary image based on observing only the pixels with a negative second-derivative intensity, and this technique is one of the useful approaches (28). In the present study, an objective lens with a high magnification was used, and the binary threshold value controls particles caused by three-dimensional effects (roughness of formulation and laser penetration). In addition, the threshold controls pixels rich in excipients, and is not suitable for being recognized as the object. Thus, the binary threshold value determines the particle agglomeration and segregation in multiple component system for image analysis, and is not necessarily 0.

In this study, the second-derivative spectrum was multiplied by−1, and the threshold value was optimized as the mean plus one half the standard deviation (mean + 0.5σ) of the specific peak intensity histogram in each area (pixels).

## RESULTS

### Characterization of Tablet Cross-sections

**Fig. 2:**
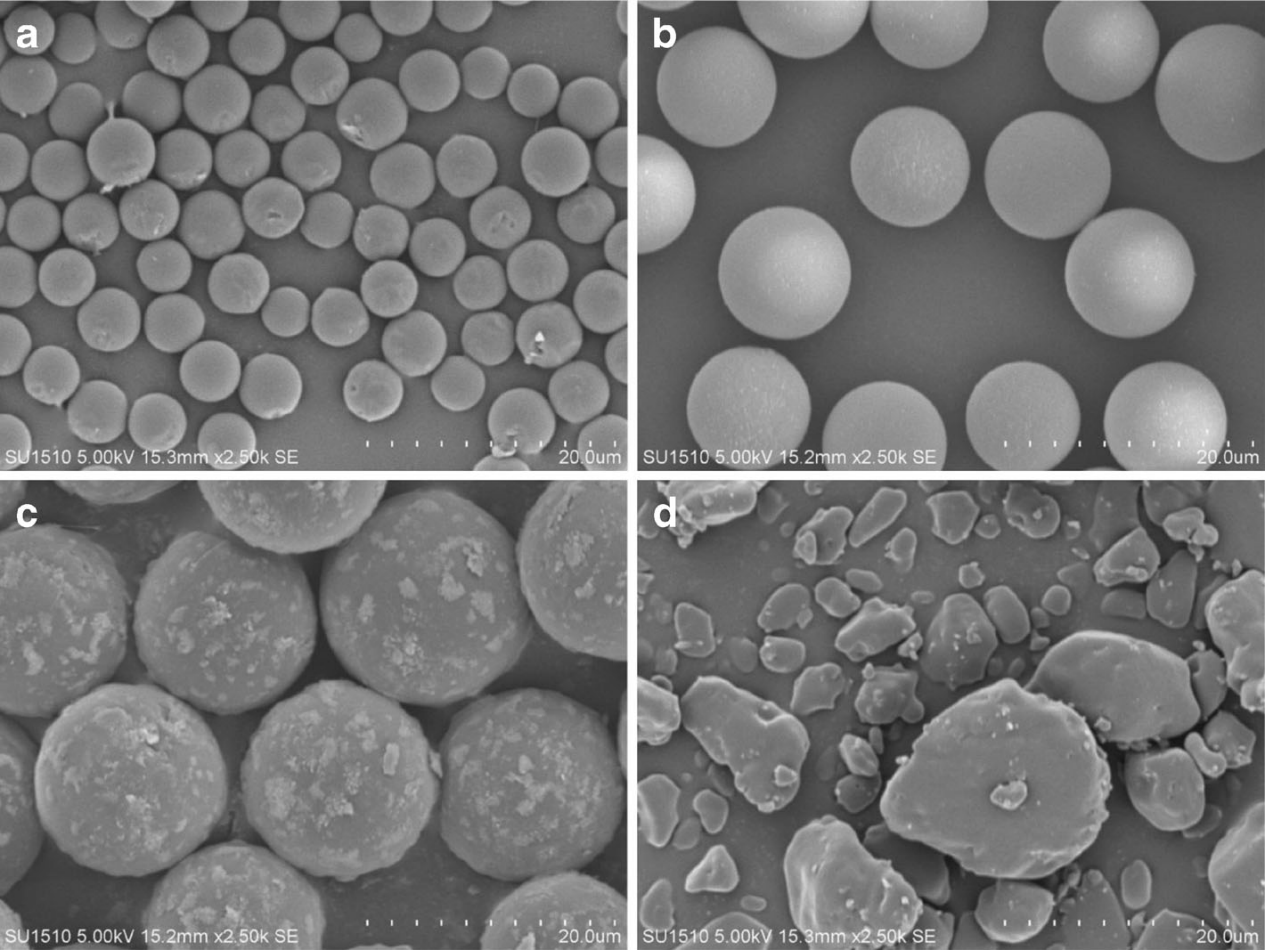
SEM micrographs of PS microspheres and ebastine. **a** 5 μm, **b** 10 μm, and **c** 15 μm PS microspheres, and **d** ebastine. *Scale bar* 20 μm

**Fig. 3:**
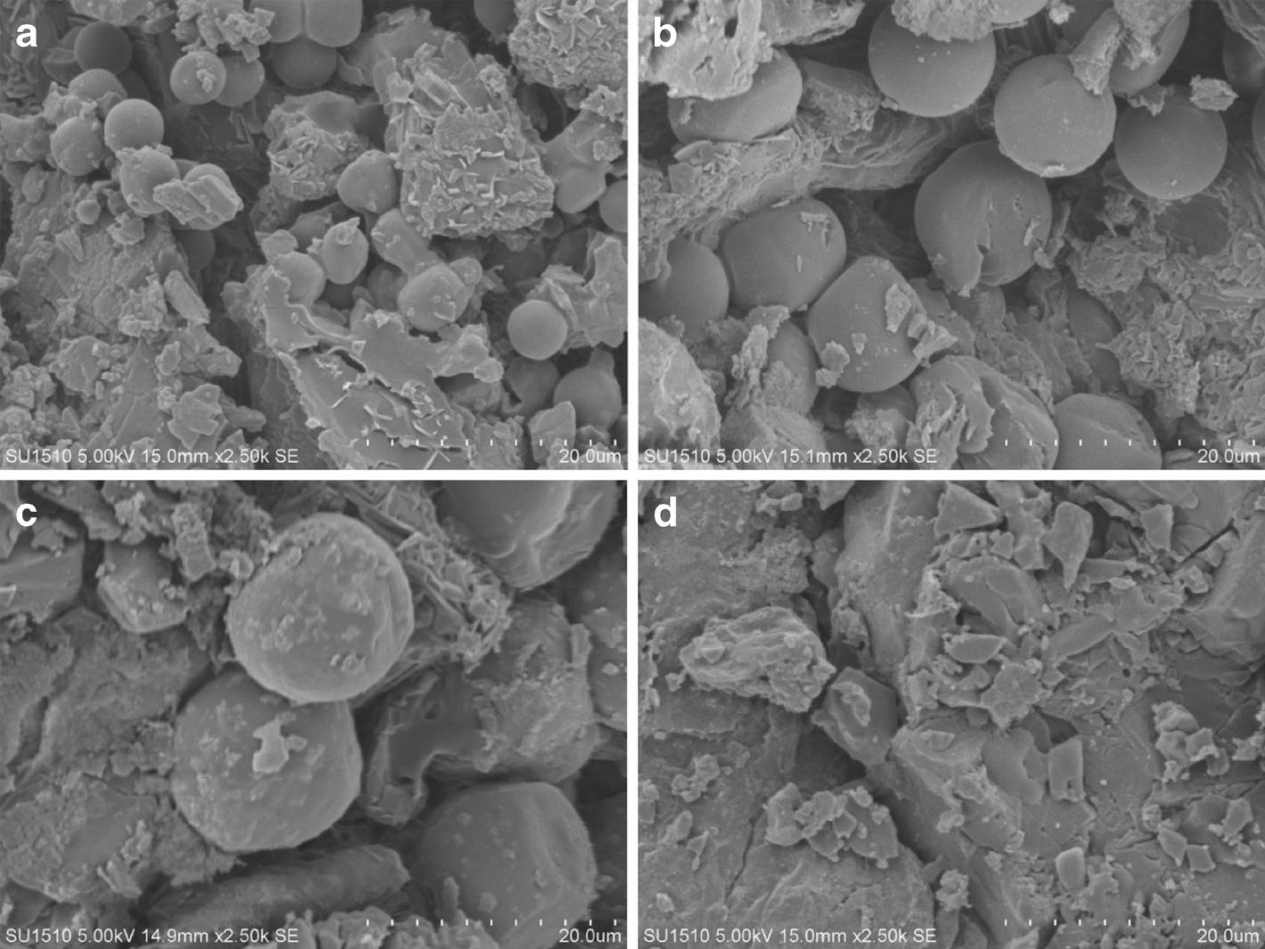
SEM micrographs of tablet cross-sections. Tablet containing **a** 5 μm, **b** 10 μm, and **c** 15 μm PS microspheres, and **d** ebastine (Ebastel tablet). *Scale bar* 20 μm

SEM and 3D confocal laser microscopy were used to visualize tablet surface profiles. Figure 2a–d shows SEM micrographs of 5, 10, and 15 μm PS microspheres and ebastine particles prior to formulation, respectively. The majority of ebastine particles in the SEM micrographs had a size distribution from 5 to 15 μm, although particles smaller than 5 μm were also observed. Figure 3a–d shows the tablet cross-sections. Micrographs indicate that the shape and size of PS microspheres in each model tablet remained unchanged. Furthermore, there were no visual differences between the ebastine particles and the excipients in SEM micrographs. Shape and size of particles were not evaluated in micrographs.

**Fig. 4:**
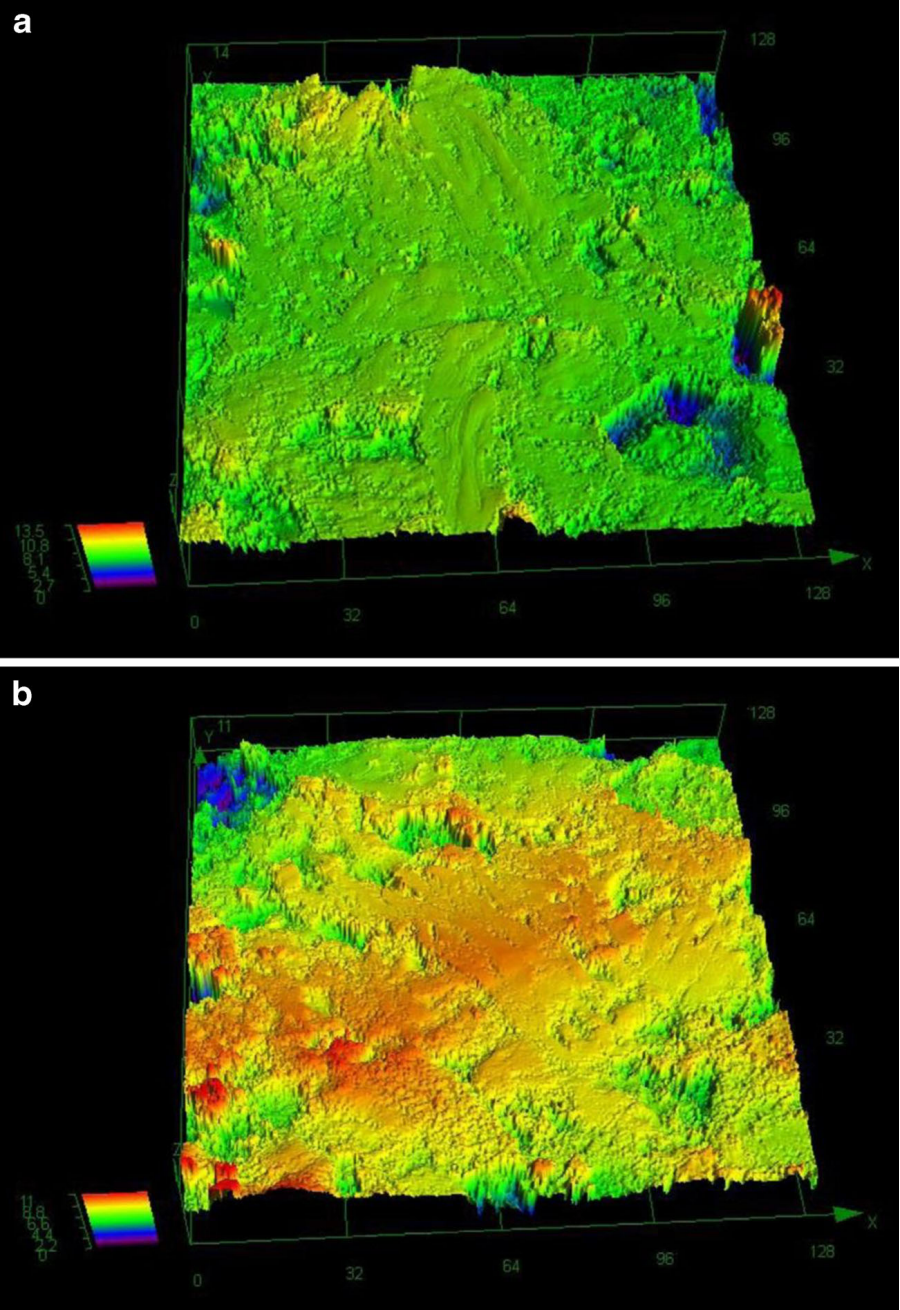
3D profiles of tablet cross-sections. Cross-sections for **a** model tablet (PS microspheres) and **b** Ebastel tablet

The 3D profiles of representative cross-sections of the model tablet and the Ebastel tablet were measured using a 3D measuring laser microscope (Fig. 4). This method is efficient because quantitative roughness can be measured. The 3D profile images indicate that the peaks and valleys from the averaged surface (yellow to green; Fig. 4) are within the range of ±10 μm in both tablets. The profiles verify that the ×50 objective used for Raman mapping allowed adequate examination of the tablet surface in this formulation.

### Application of Raman CI to Model Tablets

**Fig. 5:**
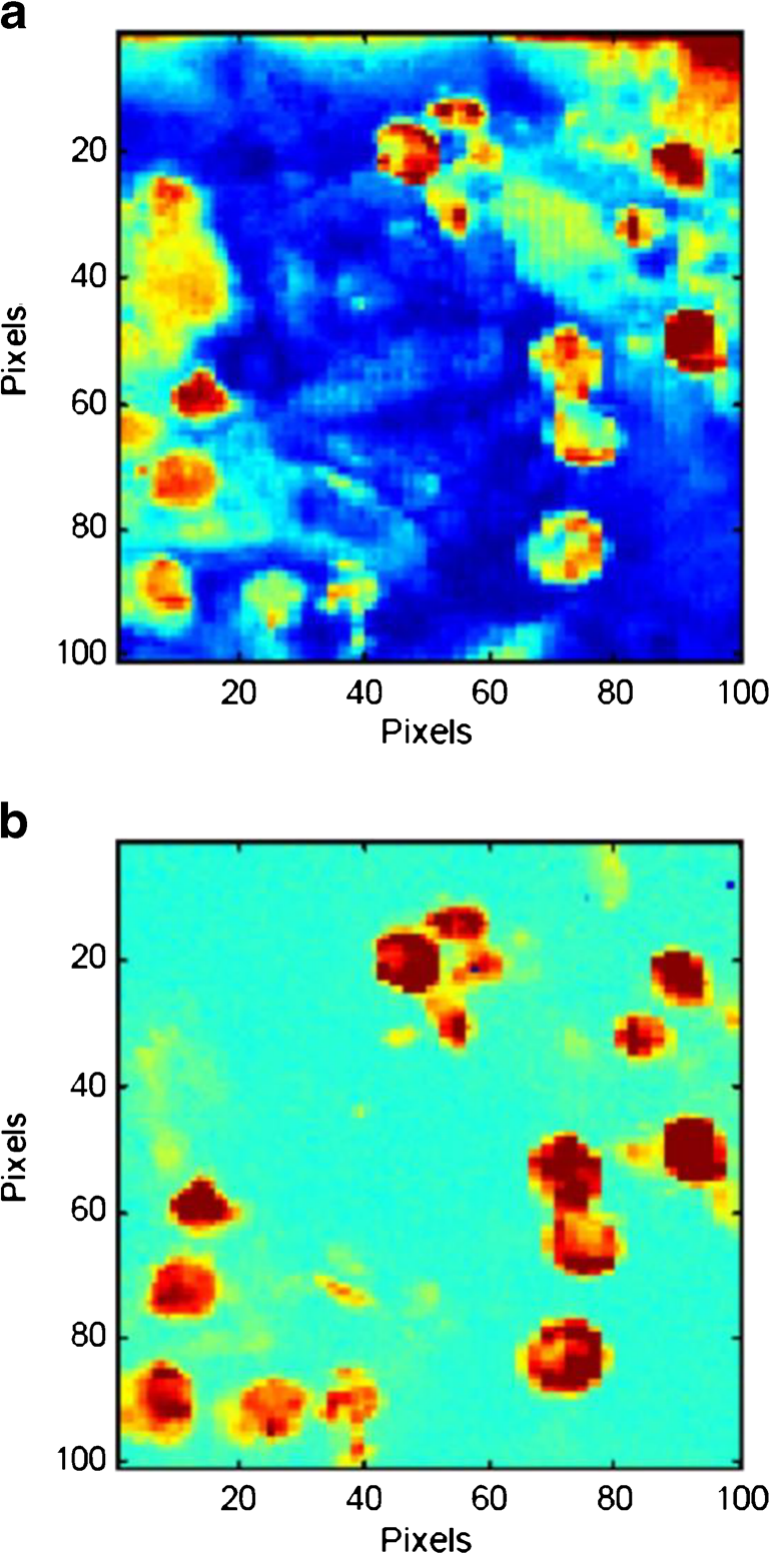
Raman chemical images of cross-section of model tablet (10 μm PS microspheres). Raman chemical images at 1,605 cm^−1^ **a** prior to preprocessing and **b** after preprocessing

**Fig. 6:**
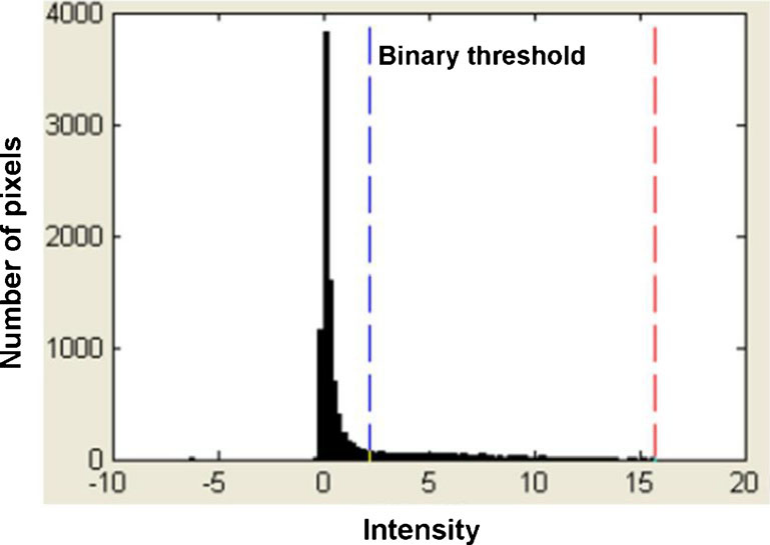
Intensity histogram at 1,605 cm^−1^ after preprocessing (10 μm PS microspheres). *Red* and *blue dashed lines* indicate maximum and threshold intensity, respectively

**Fig. 7:**
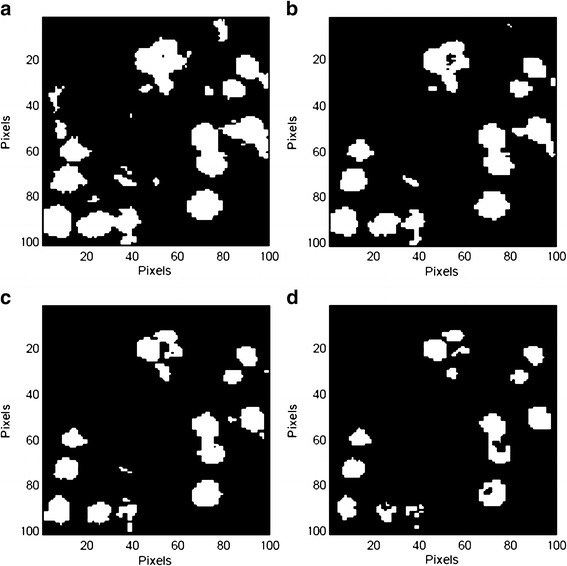
Binary images of 10 μm PS microspheres in model tablet at each threshold value. Threshold value of **a** mean, **b** mean + 0.5σ, **c** mean + 1.0σ, and **d** mean + 1.5σ in the intensity histogram

Raman CI was applied to each cross-section of the model tablets. Figure 5 shows representative Raman chemical images of the model tablet cross-section for 10 μm PS microspheres, developed based on the peak intensity of each pixel at 1,605 cm^−1^. Figure 5a is a Raman chemical image constructed using the raw Raman spectra, and Fig. 5b is the same image after second-derivative preprocessing. Figure 6 shows the intensity histogram at 1,605 cm^−1^ of the Raman chemical image after second-derivative preprocessing; Fig. 7a–d shows the binary images derived from each binarization threshold intensity in the intensity histograms: the mean, the mean + 0.5σ, the mean + 1.0σ, and the mean + 1.5σ.

### Binarization Threshold Value Determination

The appropriate binary threshold value was determined in order to accurately assess the true mean particle size of the PS microspheres in each model tablet containing individual PS microspheres, agglomerated particles, and colocalized particles in the binary images.

**Fig. 8:**
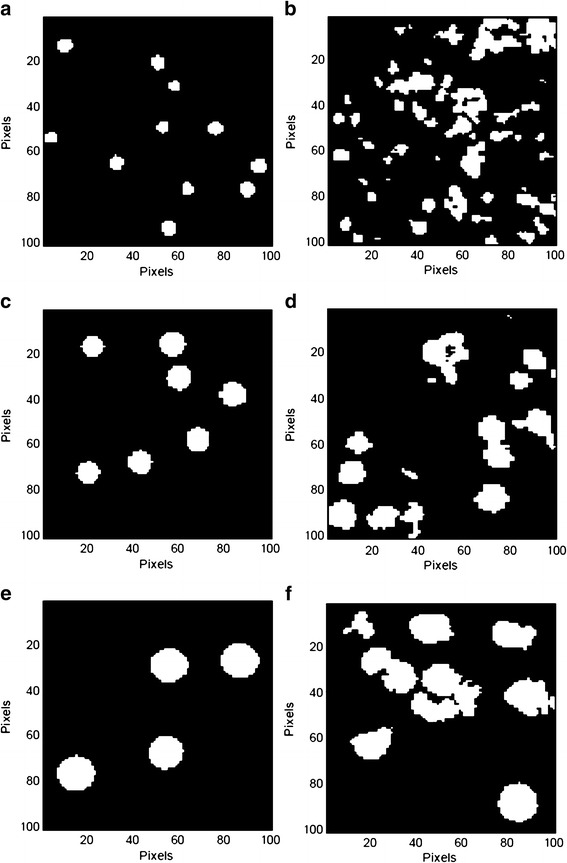
Binary images of pre- and postformulation PS microspheres; **a** 5 μm PS microspheres (preformulation), **b** 5 μm PS microspheres (postformulation), **c** 10 μm PS microspheres (preformulation), **d** 10 μm PS microspheres (postformulation), **e** 15 μm PS microspheres (preformulation), and **f** 15 μm PS microspheres (postformulation)

The threshold value of the mean + 0.5σ was applied to image analysis. The binary images of each model tablet cross-section are shown in Fig. 8, and the particle sizing results are summarized in Table I. The results for the PS microspheres prior to formulation are also shown.

**Table I:** Results for Pre- and Postformulation PS Microsphere Particle Sizing

Item	5-μm PS microspheres^a^					10-μm PS microspheres^b^					15-μm PS microspheres^c^				
	Pre	Post				Pre	Post				Pre	Post			
		1	2	3	Mean		1	2	3	Mean		1	2	3	Mean
Mean (μm)	6.30	7.07	6.36	5.74	6.20	10.58	11.17	10.16	9.81	10.19	15.79	16.42	14.15	16.96	15.90
STD (μm)	0.58	4.73	4.37	3.53	4.05	0.65	5.70	5.35	5.97	5.59	0.36	10.13	8.12	4.13	6.80
Area (%)	3.46	11.18	14.97	17.39	14.51	6.18	9.63	14.41	19.40	14.48	7.84	10.89	12.03	19.01	13.98
Particle number	11	20	32	49	101	7	8	14	19	41	4	4	6	8	18
Recovery (%)	–	112.2	101.0	91.1	98.4	–	105.6	96.0	92.7	96.3	–	104.0	89.6	107.4	100.7

*PS* polystyrene, *STD* standard deviation

^a^Certified mean particle size, 4.9 ± 0.4 μm; standard deviation, 0.5 μm

^b^Certified mean particle size, 9.8 ± 0.5 μm; standard deviation, 1.0 μm

^c^Certified mean particle size, 15.8 ± 0.6 μm; standard deviation, 1.3 μm

The recoveries (the mean particle size ratio of the post-/preformulation samples, in percent) were 98% (range, 91–112%) for the 5 μm PS microspheres, 96% (range, 93–106%) for the 10 μm PS microspheres, and 101% (range, 90–107%) for the 15 μm PS microspheres.

### Validation of Binarization Threshold Value

**Fig. 9:**
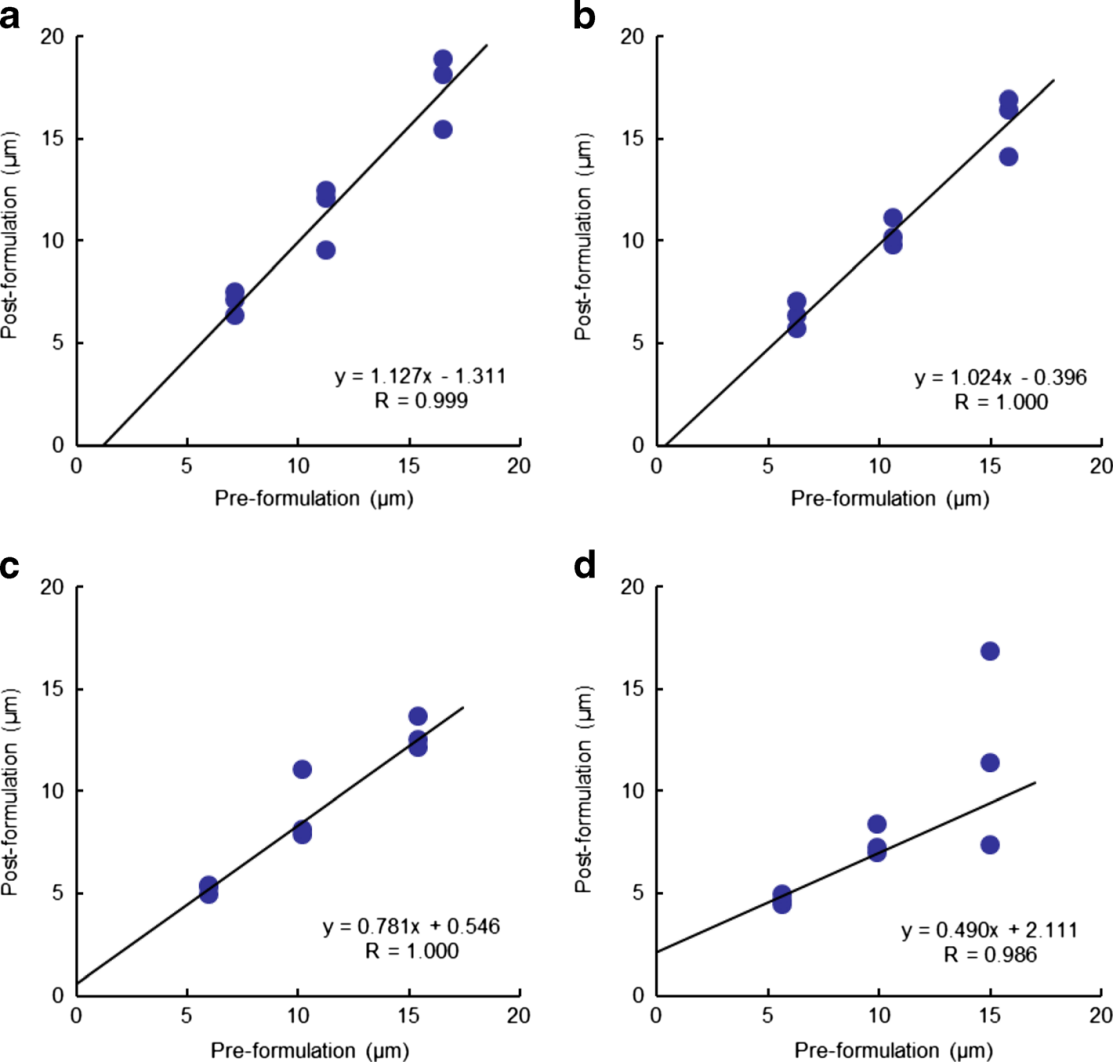
Correlations between mean PS microsphere particle size, preformulation, and postformulation. Threshold values of **a** mean, **b** mean + 0.5σ, **c** mean + 1.0σ, and **d** mean + 1.5σ. *Blue circles* indicate each result in three different areas of each tablet cross-section

Validity of binarization threshold value was tested further. Analyses were conducted at the other binarization threshold values; the mean, the mean + 1.0σ, and the mean + 1.5σ. The results were compared with those for the binarization threshold value of the mean + 0.5σ (Table II). The higher the threshold value was set, the smaller the mean particle size and the recovery in each tablet became. For the threshold value of the mean, the recoveries for the 5 and 10 μm PS microspheres were similar to those of the mean + 0.5σ; however, the recovery for the 15 μm microspheres was apparently higher than that for the mean + 0.5σ. The correlation for the mean particle size of the PS microspheres of the pre- and postformulation at each threshold value is shown in Fig. 9. The thresholds of the mean, the mean + 1.0σ, and the mean + 1.5σ, produced a regression line with a slope of 1.13, 0.78, and 0.49, respectively, whereas at the threshold value of the mean + 0.5σ, the slope was 1.02.

**Table II:** Comparison of Sizing Results for PS Microspheres at Each Threshold Value

Item	Threshold value							
	Mean		Mean + 0.5σ		Mean + 1.0σ		Mean + 1.5σ	
	Pre	Post	Pre	Post	Pre	Post	Pre	Post
5-μm PS microspheres								
Mean (μm)	7.14	6.89	6.30	6.20	5.96	5.18	5.63	4.63
STD (μm)	0.69	5.90	0.58	4.05	0.57	3.42	0.51	2.79
Area (%)	4.44	23.05	3.46	14.51	3.09	10.81	2.76	7.81
Particle number	11	107	11	101	11	106	11	102
Recovery (%)	–	96.5	–	98.4	–	86.9	–	82.2
10-μm PS microspheres								
Mean (μm)	11.26	11.11	10.58	10.19	10.15	8.50	9.87	7.39
STD (μm)	0.56	7.14	0.65	5.59	0.61	4.87	0.47	4.12
Area (%)	6.98	19.57	6.18	14.48	5.68	11.53	5.37	8.79
Particle number	7	43	7	41	7	46	7	47
Recovery (%)	–	98.7	–	96.3	–	83.7	–	74.9
15-μm PS microspheres								
Mean (μm)	16.51	17.42	15.79	15.90	15.41	12.56	14.99	9.26
STD (μm)	0.26	8.27	0.36	6.80	0.31	7.07	0.32	6.54
Area (%)	8.57	17.33	7.84	13.98	7.46	11.29	7.06	9.32
Particle number	4	18	4	18	4	21	4	28
Recovery (%)	–	105.5	–	100.7	–	81.5	–	61.8

*PS* polystyrene, *STD* standard deviation

### Application of Binarization Threshold Value to Ebastel Tablet

**Fig. 10:**
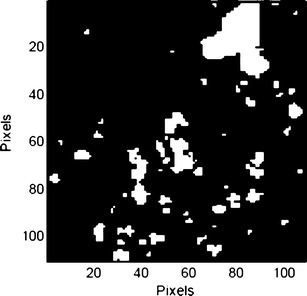
Binary image of ebastine prior to formulation

Prior to formulation, the micronized ebastine mean particle size and its standard deviation were determined using Raman CI, and were also estimated using other particle image analyzers (Morphologi G2 and FPIA-3000S). The Raman chemical image of ebastine prior to formulation is shown in Fig. 10. The results are summarized in Table III; there were no significant differences in mean particle size determined by various methods.

**Table III:** Results of Ebastine Particle Sizing Using Various Methods

Instrument	LabRAM ARAMIS	Morphologi G2	FPIA-3000S
Mean (μm)	4.11	4.11	4.71
STD (μm)	3.78	2.92	3.89
Particle number	52	10000	7725

*STD* standard deviation

**Fig. 11:**
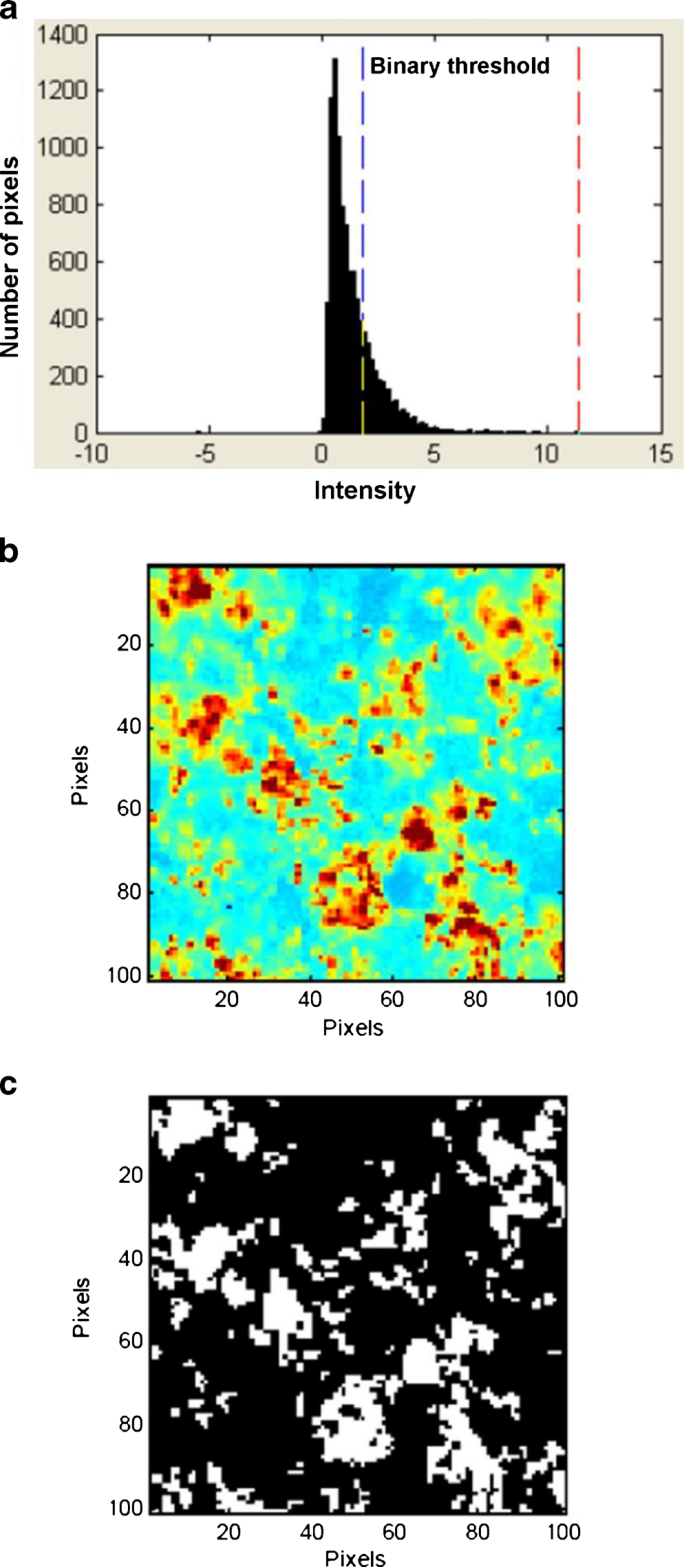
Binarization of Ebastel tablet cross-section. **a** Intensity histogram at 1,601 cm^−1^ after preprocessing. *Red* and *blue dashed lines* indicate the maximum and threshold intensity, respectively. **b** Raman chemical image of Ebastel tablet cross-section at 1,601 cm^−1^ after preprocessing. **c** Binary image of ebastine

Raman CI was used to produce a chemical image of the cross-section of an Ebastel tablet, based on the peak intensity at 1,601 cm^−1^ after second-derivative preprocessing. The resulting intensity histogram is shown in Fig. 11a. The ebastine particles could be as clearly identified as the PS microspheres in tablets using the Raman chemical image as shown in Fig. 11b. The binary threshold value of the mean + 0.5σ was applied, and the resulting binary image is shown in Fig. 11c.

Particle sizing for the three areas in the cross-section of the Ebastel tablet is summarized in Table IV. The mean size recovery (the mean particle size ratio of the post- and preformulation samples, in percent) was 105% (range, 96–112%). The relative standard deviation of the particle size was 92% for preformulation ebastine and 97% (range, 87–110%) for postformulation ebastine. The original size of the ebastine particles in the Ebastel tablets was retained during the formulation process.

**Table IV:** Results for Pre- and Postformulation Ebastine Particle Sizing

Item	Pre	Post			
		1	2	3	Mean
Mean (μm)	4.11	3.94	4.62	4.42	4.32
STD (μm)	3.78	4.34	4.35	3.85	4.17
Area (%)	10.72	22.83	24.25	22.79	23.29
Particle number	52	85	77	85	247
Recovery (%)	–	95.9	112.4	107.5	105.1

*STD* standard deviation

## DISCUSSION

### Application of Raman CI to Model Tablets

Comparison of Fig. 5a, b demonstrates that the PS microspheres were clearly identified by second-derivative preprocessing; for example, the red area in the top right corner of Fig. 5a is a region of excipients and is caused by elevation of the baseline, but is absent after preprocessing (Fig. 5b). In Fig. 5b, polystyrene particles are generally seen in only the red and yellow regions, and stable images are created. Therefore, using the second derivative, stable Raman chemical images not affected by the background spectrum can be obtained, and discrimination between the object and the background was enhanced. As shown in Fig. 6, the histogram depicts two domains clearly, and the higher peak intensity region separated by the maximum intensity and the binary threshold intensity can be taken to be the polystyrene in the tablets.

Binary images obtained using each binarization threshold intensity indicated that there were areas of separated, individual PS microspheres, as well as areas of agglomerated and colocalized PS microspheres due to particle agglomeration, tablet surface roughness, and laser penetration depth (Fig. 7). Each particle or area changed slightly for each binary threshold value. Figure 7a shows extraction of the red and yellow parts of PS microspheres in Fig. 5b, while Fig. 7d shows extraction of only the red part. Figure 7b, c show an intermediate degree of polystyrene particle agglomeration and segregation between Fig. 7a, d, respectively. Considering the shape of polystyrene particles, the binarization threshold value effectively controlled the 3D region of particles in the Raman chemical image. In addition, considering the fact that PS microspheres retained their individual shape and size during the formulation process, the threshold value determines the particle agglomeration and segregation in the binary image. While all binary images are in their true form at each threshold value, the binary threshold values for which an appropriate particle size assessment is possible are discussed below. Because the absolute value of the peak intensity varies under various conditions, it is not appropriate to define the general absolute value as the threshold value; thus, the use of statistics (*e.g.*, means, standard deviations) is useful.

### Binarization Threshold Value Determination

At the binarization threshold value of the mean + 0.5σ, the accuracy of the mean particle sizes in the tablets was good, and was independent of the particle size and area covered by PS microspheres. This suggests that the binarization threshold value and the area measured in the tablets (100 × 100 μm × 3 points) containing the micrometer-scale particles is valid for this formulation. Binary images of both particle agglomerations and colocalizations were incomplete images of individual PS microspheres, which is the limitation of image analysis with only a single binarization threshold for particle sizing. Nevertheless, a statistically reliable number of particles, which included complete individual particles, particle agglomerates, and colocalized particles in the binary images, gave a reliable mean particle size with a specific binarization threshold value. The standard deviation primarily represented the degree of particle agglomeration and segregation in the tablets.

### Validation for Binarization Threshold Value

A higher threshold value identified only parts of the PS microspheres and resulted in a mean particle size smaller than the actual size. This trend was more pronounced with larger PS microspheres prior to formulation. The threshold value of the mean produced a mean particle size larger than the actual size.

The mean particle size calculated for the PS microspheres in the tablets reflected the size prior to formulation at the binarization threshold value of the mean + 0.5σ, and good linearity and accuracy were obtained for the binarization threshold value (Fig. 9). The threshold may also work beyond the range of 5–15 μm particle size because there is no clear trend in the particle size recovery within this size range, and the threshold may be suitable even for particle size distributions. Thus, the binarization threshold value of the mean + 0.5σ created binary images that could be used to accurately estimate the mean particle size of the PS microspheres used in this formulation.

### Application of Binarization Threshold Value to Ebastel Tablet

There are strong reasons for applying this threshold value of the mean + 0.5σ to the Ebastel tablet: the Raman peak of ebastine at 1,601 cm^−1^ achieved similar intensity to that of PS microspheres at 1,605 cm^−1^ at unit exposure time, as shown in Fig. 1; the identical qualitative and quantitative excipient composition in model tablets; and the intensity histogram of an Ebastel tablet showed a similar pattern to that of polystyrene microspheres in model tablets, with the maximum intensity prior to formulation being retained. However, the binarization threshold value of the mean + 0.5σ is not a general value, and may not be suitable for other APIs.

Applying the binary threshold value of the mean + 0.5σ to the Ebastel tablet provided the mean particle sizes of ebastine in the Ebastel tablet with good accuracy. The results suggest that ebastine particles retained their size during the formulation process.

In the histogram of ebastine, it was difficult to clearly distinguish ebastine from the excipients in a certain domain. However, the binarization threshold value of the mean + 0.5σ effectively acquired the 3D region of the particles required from the unresolved distribution, and was validated in the model tablets. Some pixels with an intensity below the threshold value may have included ebastine particles of less than 1 μm in size mixed with excipients. These particles were below the quantification limit of the Raman analysis in this study, as is often the case with conventional particle sizing methods.

The concept of using model tablets for image analysis is based on the interpretation of the intensity histograms and the pixel picking method with thresholds. It may not be possible to simply apply this approach to the particular formulations that show completely different histogram patterns from that of model tablets; *e.g.*, API particles are well dispersed into the formulations and the histogram indicates no API-specific distribution, and all pixels are a mixture of components with the API maximum intensity prior to formulation not being retained (only the fact that particle size is affected by formulation process can be determined).

## CONCLUSIONS

In this study, we demonstrated that model tablets containing PS microspheres can be used to determine an appropriate binarization threshold value for assessing mean particle size of micrometer-scale components in tablets by using Raman CI. The appropriate value enabled evaluation of the influence of the formulation process on API particle size. The model tablets took advantage of the unique physicochemical properties of PS microspheres for Raman particle sizing in tablets, including the Raman spectrum, particle size, and particle shape, which remained unchanged throughout the Ebastel formulation process. The concept of model tablets containing PS microspheres as particle size standards holds promise for application to various types of formulation in the pharmaceutical industry.

## References

[1] Iacocca RG, Burcham CL, Hilden LR (2010). Particle engineering: a strategy for establishing drug substance physical property specifications during small molecule development. J Pharm Sci.

[2] Mosharraf M, Nyström C (1995). The effect of particle size and shape on the surface specific dissolution rate of micronized practically insoluble drugs. Int J Pharm.

[3] Always B, Sangchantra R, Stewart PJ (1996). Modelling the dissolution of diazepam in lactose interactive mixtures. Int J Pharm.

[4] Shekunov BY, Chattopadhyay P, Tong HHY, Chow AH (2007). Particle size analysis in pharmaceutics: principles, methods and applications. Pharm Res.

[5] Randall CS, Brittain HG (1996). Particle size distribution. Physical characterization of pharmaceutical solids.

[6] Shekunov BY, Feeley JC, Chow L, Tong HHY, York P (2003). Aerosolisation behaviour of micronised and supercritically-processed powders. J Aerosol Sci.

[7] Haskell RJ (1998). Characterization of submicron systems *via* optical methods. J Pharm Sci.

[8] Bosquillon C, Lombry C, Preat V, Vanbever R (2001). Comparison of particle sizing techniques in the case of inhalation dry powders. J Pharm Sci.

[9] Kaye BH, Alliet D, Switzer L, Turbitt-Daoust C (1997). The effect of shape on intermethod correlation of techniques for characterizing the size distribution of powder. Part 1: correlating the size distribution measured by sieving, image analysis and diffractometer methods. Part Part Syst Charact.

[10] Kaye BH, Alliet D, Switzer L, Turbitt-Daoust C (1999). The effect of shape on intermethod correlation of techniques for characterizing the size distribution of powder. Part 2: correlating the size distribution as measured by diffractometer methods, TSI-Amherst aerosol spectrometer and Coulter counter. Part Part Syst Charact.

[11] Corcoran TE, Hitron R, Humphreys W, Chigier N (2000). Optical measurement of nebulizer sprays: a quantitative comparison of diffraction, phase Doppler interferometry and time of flight techniques. J Aerosol Sci.

[12] Shekunov BY, Chattopadhyay P, Seitzinger J, Huff R (2006). Nanoparticles of poorly water-soluble drugs prepared by supercritical fluid extraction of emulsions. Pharm Res.

[13] Mitchell JP, Nagel MW, Wiersema KJ, Doyle CC (2003). Aerodynamic particle size analysis of aerosols from pressurized metered-dose inhalers: comparison of Andersen 8-stage cascade impactor, next generation pharmaceutical impactor and model 3321 aerodynamic particle sizer aerosol spectrometer. AAPS PharmSciTech.

[14] Ely D, Chamarthy S, Carvajal MT (2006). An investigation into low dose blend uniformity and segregation determination using NIR spectroscopy. Colloids Surf A Physicochem Eng Asp.

[15] Aaltonen J, Gordon KC, Strachan CJ, Rades T (2008). Perspective in the use of spectroscopy to characterize pharmaceutical solids. Int J Pharm.

[16] Wartewig S, Neubert RHH (2005). Pharmaceutical applications of mid-IR and Raman spectroscopy. Adv Drug Deliv Rev.

[17] Pöllänen K, Häkkinen A, Reinikainen SP, Rantanen J, Karjalainen M, Louhi-Kultanen M (2005). IR spectroscopy together with multivariate data analysis as a process analytical tool for in-line monitoring of crystallization process and solid-state analysis of crystalline product. J Pharm Biomed Anal.

[18] Roggo Y, Chalus P, Maurer L, Lema-Martinez C, Edmond A, Jent N (2007). A review of near infrared spectroscopy and chemometrics in pharmaceutical technologies. J Pharm Biomed Anal.

[19] Rantanen J (2007). Process analytical applications of Raman spectroscopy. J Pharm Pharmacol.

[20] Davis KL, Kemper MS, Lewis IR, Šašić S (2008). Raman spectroscopy for monitoring real-time processes in the pharmaceutical industry. Pharmaceutical applications of Raman spectroscopy.

[21] Gendrin C, Roggo Y, Collet C (2008). Pharmaceutical applications of vibrational chemical imaging and chemometrics: a review. J Pharm Biomed Anal.

[22] Šašić S, Šašić S (2008). Raman chemical imaging of solid dosage formulations. Pharmaceutical applications of Raman spectroscopy.

[23] Wessel E, Vogel C, Kolomiets O, Hoffmann U, Siesler HW, Salzer R, Siesler HW (2009). FT-IR and NIR spectroscopic imaging: principles, practical aspects and applications in material and pharmaceutical sciences. Infrared and Raman spectroscopic imaging.

[24] Lee E, Salzer R, Siesler HW (2009). Raman spectral imaging on pharmaceutical products. Infrared and Raman spectroscopic imaging.

[25] Slipchenko MN, Chen H, Ely DR, Jung Y, Carvajal MT, Cheng JX (2010). Vibrational imaging of tablets by epi-detected stimulated Raman scattering microscopy. Analyst.

[26] Hilden LR, Pommier CJ, Badawy SIF, Friedman EM (2008). NIR chemical imaging to guide/support BMS-561389 tablet formulation development. Int J Pharm.

[27] Ely DR, Carvajal MT (2011). Determination of the scale of segregation of low dose tablets using hyperspectral imaging. Int J Pharm.

[28] Jérez Rozo JI, Zarow A, Zhou B, Pinal R, Iqbal Z, Romaňach RJ (2011). Complementary near-infrared and Raman chemical imaging of pharmaceutical thin films. J Pharm Sci.

[29] Šašić S, Zhang L, Šašić S, Ozaki Y (2008). Pharmaceutical application of Raman chemical imaging. Raman, infrared, and near-infrared chemical imaging.

[30] Henson MJ, Zhang L (2006). Drug characterization in low dosage pharmaceutical tablets using Raman microscopic mapping. Appl Spectrosc.

[31] Doub WH, Adams WP, Spencer JA, Buhse LF, Nelson MP, Treado PJ (2007). Raman chemical imaging for ingredient-specific particle size characterization of aqueous suspension nasal spray formulations: a progress report. Pharm Res.

